# Females at a Clear Disadvantage with Postoperative Myocardial Infarction Symptoms

**DOI:** 10.3390/jcdd11110371

**Published:** 2024-11-19

**Authors:** Sonja Guethoff, Rebekka Kraft, Matthias Riege, Carola Grinninger, Kara Krajewski

**Affiliations:** 1 School of Health and Social Sciences, AKAD University, 70191 Stuttgart, Germany; sonja.guethoff@akad.de (S.G.); rebekka.kraft@stud.akad.de (R.K.); 2 School of Engineering and Technology Management, AKAD University, 70191 Stuttgart, Germany; matthias.riege@akad.de; 3 Clinic of Cardiovascular Surgery, University Munich, 81377 Munich, Germany

**Keywords:** gender medicine, clinical vignettes, myocardial infarction, gender bias, gender blindness

## Abstract

Background: Cardiovascular disease remains the leading cause of death in women. Gender bias and blindness are coming into focus as relevant risk factors for patients. To date, there have been no studies that target surgeons’ potential gender bias in recognizing postoperative myocardial infarction (MI). Methods: An online clinical vignette describing a patient with postoperative MI was randomized for gender. Possible diagnoses, the next steps in management, and questions on gender bias were assessed. Results: A total of 205 surveys were analyzed. MI was recognized in 55.6% of the male case studies vs. 32.0% of the female case studies in the first question (*p* < 0.005). Cardiac diagnostics were initiated significantly more in male case studies (94% vs. 76%, *p* = 0.001). Female surgeons listed MI as the first diagnosis twice as often as male surgeons overall (43% vs. 23%, *p* = 0.027). Female surgeons were also more likely to mention MI across the survey at all compared to male surgeons (89% vs. 67%, *p* = 0.0002). Board-certified surgeons diagnosed MI by the end of the survey significantly more (88.2%) than residents (75.0%) and medical students (75.0%, *p* = 0.047). Conclusions: Overall, this study was able to demonstrate the presence of both gender bias and gender blindness in surgeons’ assessment of postoperative myocardial infarction symptoms with a clear disadvantage for female patients and a superior awareness for female surgeons.

## 1. Introduction

Cardiovascular disease remains the leading cause of death in women in the U.S. [1], and more women than men die from cardiovascular disease across the European Union [2]. In managing atherosclerotic cardiovascular disease in women, studies report reduced symptom recognition, longer pre-hospital delays, and prolonged times to initial EKG, cardiology consultation, and door-to-balloon intervention upon hospital arrival [3]. In a recent study cohort including all patients after major cardiac surgery, women presented with a significantly higher age and considerably more frequently with severe cardiac impairment (NYHA III or IV) [4], which may suggest a later diagnosis of their cardiovascular disease. It has also been shown that female gender is an independent risk factor for worse survival after cardiosurgical procedures [5,6].

One approach is that the biological risk factor profile in postmenopausal women is responsible for these findings, as highlighted in a recent review with respect to the cellular and even chromosomal level, and affects cardiac remodeling at all phases of life [7]. It is also possible that other factors such as poorer communication with healthcare representatives and a lack of consideration of sex-specific and gender-specific pathophysiology in medical research; moreover, the underrepresentation of females in drug studies [8] resulting in an overall lack of knowledge is to blame, otherwise known as “gender blindness” [9]. Integrating sex and gender variables into research is essential to address historical knowledge gaps, reduce health disparities, and promote equitable healthcare outcomes, thereby advancing public health through more rigorous, personalized, and inclusive scientific practices [10,11]. Public awareness in gender-related cardiovascular disease is increasing, yet the American Heart Association did not issue its first female-specific clinical recommendations for the prevention of cardiovascular disease until 1999 [1]. Others have shown that gender bias exists with respect to pain in both a retrospective clinical setting and a clinical vignette setting [12,13,14]. Studies utilizing video vignettes on coronary heart disease patients have also shown that gender bias exists among primary care physicians in several countries [15,16].

Postoperative myocardial infarction (MI) is a possible complication encountered after various surgical procedures, and to date there have been no studies that target surgeons’ potential gender bias in this context. Thus, the goal of this study was to assess whether there were gender-related differences in the recognition of postoperative myocardial infarction in an online clinical vignette survey among surgeons in Germany.

## 2. Materials and Methods

The study was reported to the local ethics committee as per protocol. Ethical review and approval were waived for this study due to local ethics regulations. A clinical case vignette for a 62-year-old patient with typical cardiovascular risk factors (i.e., smoking, hypertension, obesity) presenting with postoperative pain indicative of a myocardial infarction was developed by the senior physician team comprising the areas of cardiology, cardiothoracic surgery, and neurosurgery (see Appendix A). An online survey containing either the male and female case study was posted at www.umfrageonline.com, accessed on 23 September 2024. The randomized equally distributed assignment of the participants to one of the two case studies was carried out automatically using a PHP script on a Web server. The cases and questions were exactly the same except for the patient’s gender. The symptom of nausea was also deliberately included in order to correspond to female symptoms. Over the process of the query, further information wase given to lead the participant to the correct diagnosis. After revealing the diagnosis, further questions assessed the phenomenon of underestimating MI in female patients. See Appendix A for the full (translated) survey.

The link for the survey was shared via the largest German network of female surgeons (Chirurginnen e. V., Adelheidsdorf, Germany) at multiple time points. In addition, various surgical departments of both university and non-university clinics throughout Germany were contacted via email to the chief of surgery or his/her secretary (n = 26 clinics). Data were collected between May 21st and July 23rd 2024.

The first three questions assessed the participants with respect to gender, experience, and surgical discipline. The presence of “myocardial infarction” or a related term was assessed in the following three questions. Next, three options were provided for the following question about the next action to be taken, to ensure that the decision for the next step is not obvious; both choices with “reassurance” instead of “initiate diagnostics” were then grouped together for further analyses. For the next question in which the participant lists the intended diagnostics, clear cardiovascular parameters (i.e., “heart enzymes” and not “draw labs”) were considered correct. The participant was then given the information that the patient had cold sweats and was short of breath; another open-ended question for the next steps followed. The participants were then asked if they underestimate M.I. in women in their clinical practice (yes/no format). The last two questions were open-ended on the topic of possible causes of underestimating MI in women and how this phenomenon can be counteracted. The two senior authors (S.G., K.K.) independently verified the categories of answers for the final open-ended questions and then agreed on the end results.

Statistical analyses were performed with SPSS (Version 29.0.2.0 IBM Corp., Armonk, NY, USA). A *p* value less than 0.05 was considered significant for the main hypotheses of the study; the remaining analyses were considered explorative. Fisher’s exact test was performed due to the small sample sizes. The null hypothesis was that there would be no difference between the rate of detection or time of detection of the myocardial infarction in the male and female cases. Further, there would be no differences between detection rates or time of detection between male and female participants.

## 3. Results

### 3.1. General Descriptive Statistics on the Survey

A total of 2.465 surveys were started. 266 were begun (186 for the male case study, 80 for the female case study). Therefore, the participation rate was 10.8%, with a considerable preference to answer the male patient case study (13.9% vs. 7.1% for the female patient case study, *p* < 0.00001). Surveys that included an answer to the first case-related question were included in further analyses. Five participants had to be excluded from further analyses as they were not part of a surgical specialty. A total of 205 cases were thus included in further analyses (n = 146 or 71.2% cases with the male patient case study and n = 59 or 28.8% cases with the female patient case study). Therefore, 81% (n = 166) answered the questions pertaining to the case study completely (82.8% for the male case, 76.3% for the female case).

### 3.2. Demographics of Survey Participants

**Figure 1:**
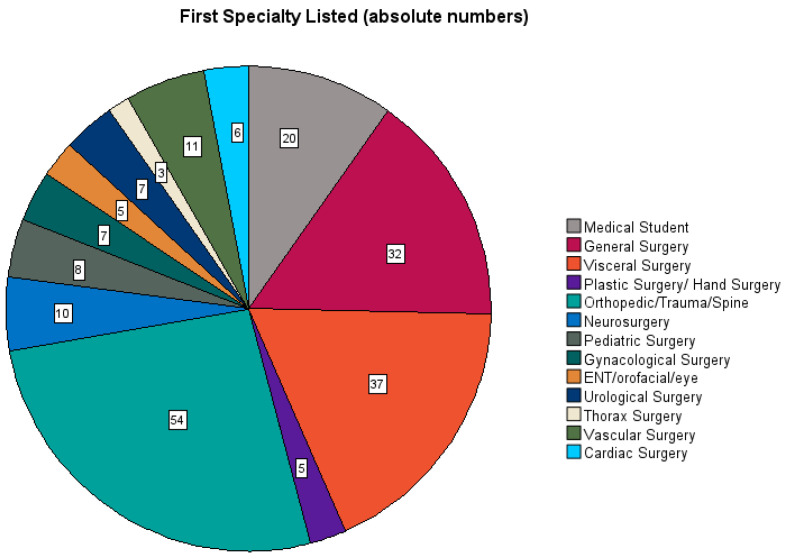
Representation of the surgical specialties among survey participants.

In the included cohort, 79.5% (n = 163) of the participants were female, 20.5% (n = 42) were male; the gender “diverse” was not selected. Overall, 26.8% were resident physicians in surgery and 63.4% were board-certified surgeons, summarized together as the cohort of surgeons (male surgeons n = 40 and female surgeons n = 145). The sub-cohort of medical students was small at 9.8% (male n = 2 and female n = 18). This sub-cohort of students was separated for a final analysis in order to evaluate a trend as to whether gender medicine is better addressed by the current medical degree program. Among the physicians and surgeons, orthopedic/trauma/spine surgery was the most frequent specialty (26.3%), followed by visceral surgery (18%) and general surgery (15.6%). Cardiovascular surgeons made up 8.3% of participants. See Figure 1.

### 3.3. Analyses of the Survey Content in the Cohort of Surgeons 

The first question asked in general terms what was going through the participants’ minds as they were woken up by nursing staff during a night shift. The subsequent questions increasingly referred to a specific diagnosis and corresponding diagnostics. More clinical symptoms were given in the process of the study in order to increasingly lead to the diagnosis of myocardial infarction. By the end of the survey, the diagnosis “myocardial infarction” or a related term (i.e., acute coronary syndrome, heart attack, etc.) was provided in 114/133 cases (85.7%) in the male patient case study and in 37/46 (80.4%) cases in the female patient case study (*p* = ns).

**Figure 2:**
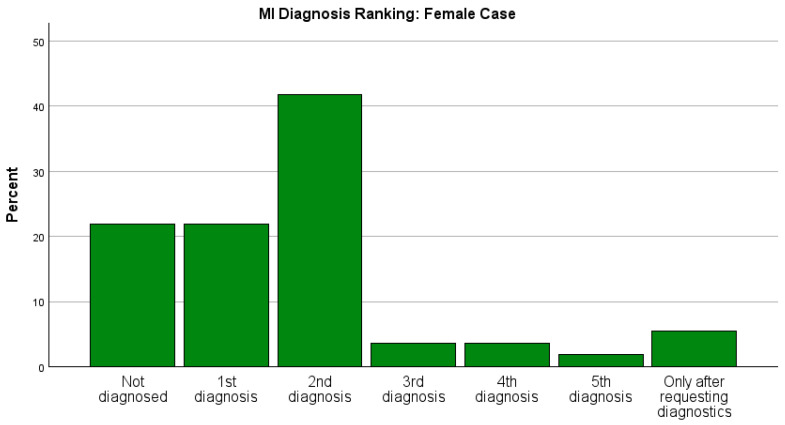

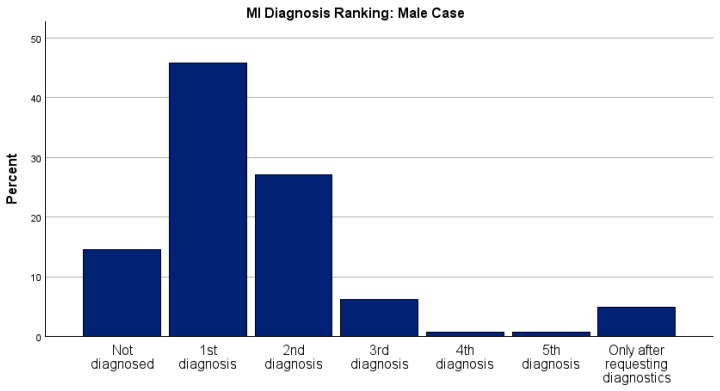
A graphic representation of where MI was mentioned for the female and male case studies, including all participants of the survey. The rate of detection as a first diagnosis between the male and female case was significant (*p* = 0.003).

Therefore, the time and rank at which myocardial infarction was considered as a diagnosis in the two case studies was of interest in this investigation. Both parameters showed a clear difference between the two case studies. Already in the first question, the diagnosis “myocardial infarction” or a related term was listed for a total of 91 of 185 participants (49.2%); however, it was listed for (75/135) 55.6% of participants for the male case study and only (16/50) 32.0% of participants for the female case study (Fisher’s exact test, *p* < 0.005). Across the whole survey, myocardial infarction was named as the first diagnosis by 60/133 (45.1%) of participants in the male patient case study versus 10/46 (21.7%) in the female patient case study (*p* = 0.003), including cases where the diagnosis was only made after “prompting” from the further questions of the survey. The distribution of the place where the diagnosis was mentioned in both case studies is shown in Figure 2.

Regardless of case gender, female surgeons listed “myocardial infarction” or a related term as the first diagnosis twice as often as male surgeons (43% vs. 23%, *p* = 0.027). Female surgeons were also more likely to mention “myocardial infarction” or a related term across the survey overall compared to male surgeons (89% vs. 67%, *p* = 0.0002). In the survey, 35.7% of female surgeons admitted to underestimating myocardial infarction in female patients compared to 18.2% of male surgeons.

Overall, the male patient case study was more likely to have diagnostics vs. reassurance compared to the female patient case study (94% vs. 76%, *p* = 0.001). Notably, male surgeons suggested “diagnostics” in 100% of cases in the male patient case study compared to 79% of cases in the female patient case study. Cardiac diagnostics were also listed explicitly more often in the male patient case study compared to the female patient case study (88% vs. 68%, *p* = 0.005).

### 3.4. Analyses Depending on the Level of (Further) Training

**Figure 3:**
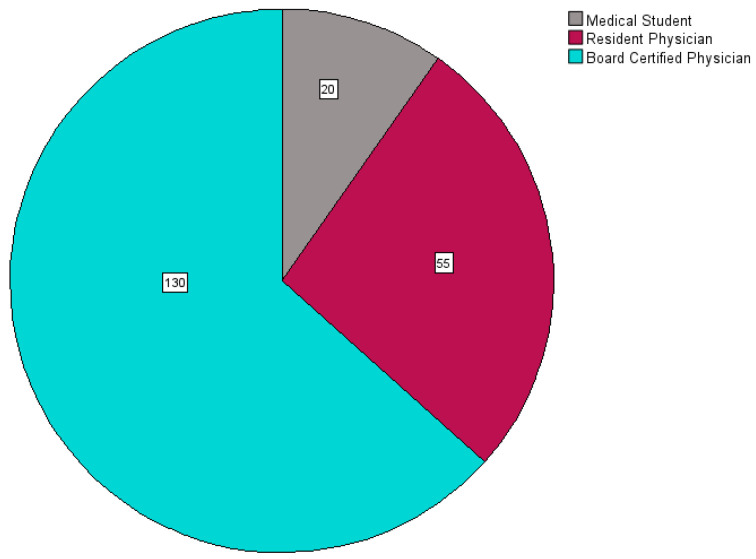
Participants’ level of training (in absolute numbers).

The majority of participants were board-certified physicians. See Figure 3.

**Figure 4:**
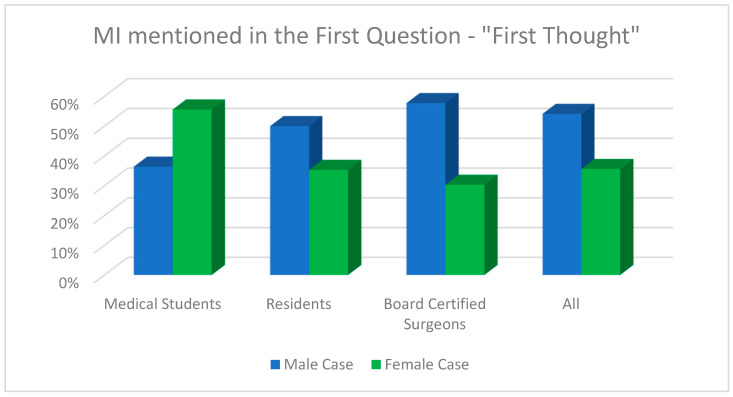
Response of MI within the first answer field for each case according to participants’ experience.

**Figure 5:**
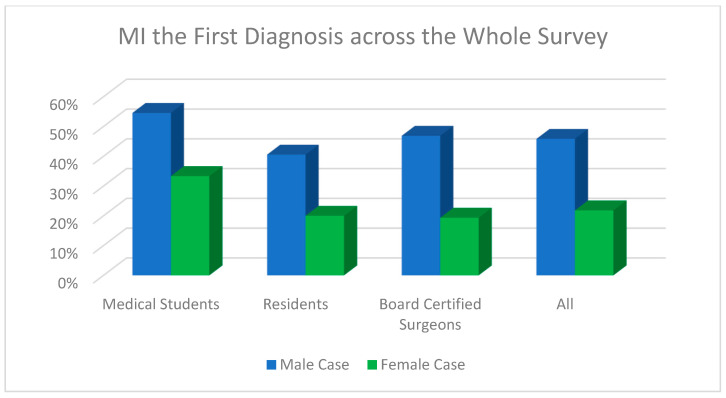
MI as the first diagnosis across the whole survey for each case according to participants’ experience.

**Figure 6:**
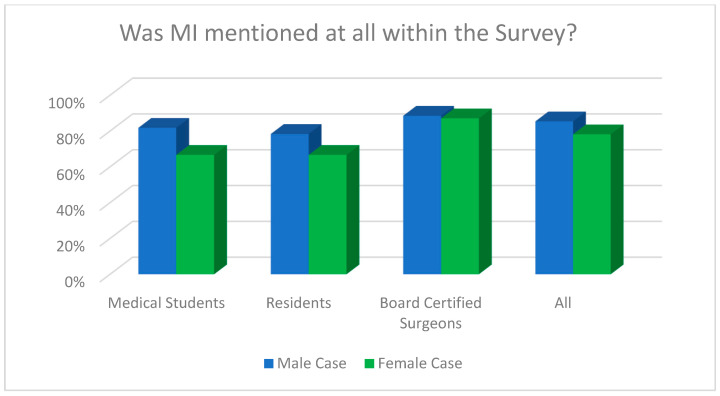
An analysis of whether MI was mentioned as a diagnosis across the whole survey for each case according to participants’ experience.

Regarding the question whether myocardial infarction was diagnosed by the end of the survey overall (for both cases), there was a statistically significant difference between board-certified doctors (88.2%) compared to residents (75.0%) and medical students (75.0%, Fisher’s exact test, *p* = 0.047). Figure 4, Figure 5 and Figure 6 demonstrate the answers to the key survey questions according to participants’ experience.

### 3.5. Why Is MI Underestimated in Female Patients?

**Figure 7:**
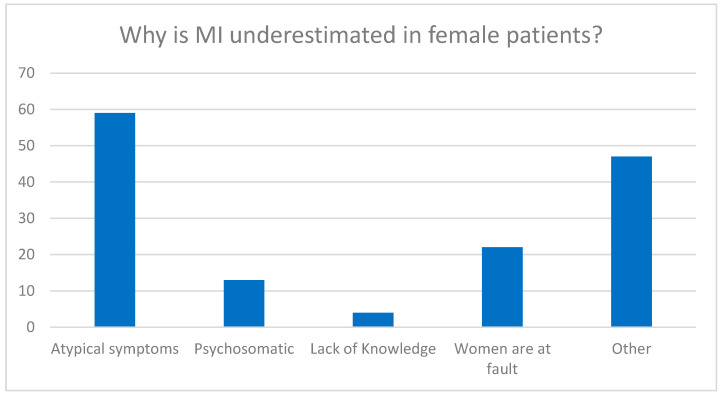
Multiple answers evaluated from the open-ended survey question.

This open-ended question was answered by n = 203 participants. When asked why myocardial infarction in women could be underestimated by medical colleagues, the most common answer was that the symptoms in women were “atypical” (n = 72/150, 48.0%) or different from the “classic” symptoms found in men. It was also mentioned by 22 participants that women would more often play down the situation and, for example, report less pain, i.e., that it was basically their own fault (see Figure 7).

How can we improve awareness? This open-ended question was answered by n = 159 participants. 16.4% answered “no idea”; 73.6% mentioned forms of “education”; 10.1% “other” (i.e., “change textbooks to include female case studies on ACS”, “hope the next generation is more aware”).

## 4. Discussion

Overall, this study was able to demonstrate the presence of both gender bias and gender blindness in surgeons’ assessment of postoperative myocardial infarction symptoms with a clear disadvantage for the female case study.

The first significant finding was the preference for participants to finish the male case study compared to the female case study; twice as many male case studies were finished. Further, the gender distribution of participants was not representative, with nearly 80% being female surgeons, which is possibly a reflection of the media used to recruit participants. According to the German Federal Board of Physicians, as of 2023, the ratio of male to female surgeons receiving board certification was 3:1 in the majority of surgical specialties [17]. As the group of male surgeons was thus smaller and not representative of the situation in Germany as a whole, few analyses according to surgeon gender could be made. However, the primary focus of the study was on the patient’s gender in the case study.

There was indeed a significant difference between listing MI as a diagnosis in the first question of the vignette in favor of male patients (56% vs. 32% in the female vignette). Across the survey as a whole, MI was listed as the first diagnosis twice as often in the male case than in the female case. Cardiac diagnostics were also ordered significantly more often in the male case study. These findings are similar to studies on primary care physicians with video vignettes on coronary heart disease which also discovered that female case studies received fewer exams, fewer cardiac diagnostics, and different management compared to male patients [15,16].

It is not only physicians who seem to underestimate coronary heart disease in women. One study group examined gender bias in bystander/first responder rates in North Carolina from 2010 to 2014 after a campaign to increase education on basic and advanced CPR, and found that female cardiac arrest patients had a longer time from collapse to first defibrillation use compared to male patients, as well as a significant improvement in male patients’ survival and neurological outcome after the educational campaign compared to female patients [18]. A similar study in Sweden in 2024 found lower 30-day survival rates for women after out of hospital cardiac arrest [19]. Contributing factors were lower percentages of shockable rhythms comparable to men, as well as older age in female patients and lower income [19]. It was also found that women had lower rates of bystander defibrillation or defibrillation by EMS first responders than male patients [19].

Female patient status has also been found to be associated with grave non-cardiac consequences. For example, female patients treated by male surgeons had the worst postoperative outcome (i.e., death, complications with readmission) after undergoing common surgical procedures in a surgeon–patient gender study [20]. An older study from 1993 on primary care physicians in the American Midwest and their rates of ordering Pap smears and mammograms in female patients different significantly according to physician gender; female doctors were twice as likely to order Pap smears than their male colleagues and 1.4x as likely to order mammograms than their male colleagues [21]. An older study on analgesic use in outpatient cancer patients with metastases found that female gender was also a risk factor in not receiving adequate analgesics [14]. A medical vignette study, similar to the present study, was carried out in the USA among primary care physicians to assess the prescription rates of opioid analgesics in low back pain according to patient race and gender [13]. They found that physicians were more likely to prescribe opiates to same-sex patients with back pain [13]. A German group prospectively examined the reported pain rates after open heart surgery depending on the gender of the examiner and found that higher pain levels were reported significantly more to female assessors for both genders despite equal levels of pain reported; this effect was more pronounced in male patients [12]. Thus, physician gender also seems to play a key role in patient care.

The effect of physician gender on women’s health is highly pronounced in surgical fields, as mentioned above [15]. A similar study found a lower rate of adverse postoperative outcomes including mortality at 90 days and 1 year was also found in favor of female surgeons, especially female surgeons treating female patients [22]. A large American study on over 2 million patients over 65 undergoing major surgeries found that the lowest postoperative mortality rates were best in the female surgeon–female patient combination; this study also had an interesting side finding, namely that female surgeons were more likely to treat patients with serious conditions (i.e., higher association with death) than male surgeons [22]. The cohort of female surgeons was much smaller than male surgeons and still a trend of less mortality was found in the female surgeon–female patient tandem [22]. A recent Canadian study on over 700,000 patients undergoing major inpatient procedures examined postoperative 90-day major morbidity and found a significantly lower odds ratio of major morbidity within teams with more females; in fact, these findings were even more pronounced when the anesthesiologist and/or surgeon were, in fact, female [23]. We were not able to perform meaningful statistical analyses between male and female surgeons among our participants for every question due to the small sample size of male participants, as mentioned above. However, we were able to note that female surgeons were twice as likely to mention MI than male surgeons. We also found that male surgeons chose to initiate diagnostics in all patients in the male case instead of only offering reassurance, which was their next course of action for only 79% in the female case. Further, roughly twice as many female surgeons as male surgeons admitted to underestimating myocardial infarction in female patients, even though participants were blinded to the gender randomization behind the vignette. In light of the present study, it is possible that perioperative care is a major determinant in surgical outcome studies and should be the target of future clinical investigations.

When asked why CHD is underestimated in women, 48% of participants in the current study (across genders) responded that symptoms are “atypical” in women. It seems odd that symptoms for 50% of the world’s population are considered “atypical”. The most recent European Society of Cardiology (ESC) guidelines for the management of acute coronary syndrome from 2023 actually no longer differentiate between “male vs. female” or “typical vs. atypical” symptoms but rather describe the most frequent symptoms and urge clinicians to use the algorithm “cardiac” vs. “possibly cardiac” vs. “likely non-cardiac” symptoms [24].

Nearly three-quarters of participants advocated for more education on gender sensitivity to increase the awareness of CHD in female patients. Few studies on the topic exist; one recent study that assessed gender sensitivity and stereotypes among Swiss medical students found more gender stereotypes for male students compared to females [25]. Further, they found that gender sensibility increased with increasing student age and gender stereotypes decreased with increasing student age [25]. That university had implemented gender medicine lectures into medical school curriculum as early as 2005 [25].

When assessing MI listed as a diagnosis by the end of the survey according to participants’ experience, the present study found board-certified surgeons to significantly detect the correct diagnosis compared to residents and medical students. Thus, experience is also a good predictor for a correct diagnosis. However, an obligatory gender medicine curriculum should be included in medical training to improve the knowledge of all physicians.

## 5. Conclusions

This survey demonstrates a clear underestimation of postoperative myocardial infarction/cardiovascular risk in women. Although the present study was not representative with respect to surgeon gender distribution with a much higher participation rate of female surgeons, it could be shown that female surgeons were more likely to diagnose postoperative MI overall than male surgeons. Regarding the question whether myocardial infarction was diagnosed by the end of the survey overall (for both cases), there was a statistically significant difference in favor of board-certified doctors compared to residents and medical students. Symptoms of MI in women, who make up about half of the world´s population, are still widely falsely perceived as “atypical”. More targeted education of cardiovascular risk and clinical presentation is needed to counteract gender bias and blindness and to improve outcome in female patients. It is desirable that there is a mind shift in medicine so that the symptoms experienced by half of humanity are not considered “atypical” anymore.

## Data Availability

Raw data available upon request.

## Appendix A

### Translation of the Original German Case Study

Imagine you are on night shift, on call or back-up call: A 62-year-old business (wo) man repeatedly complains of pain in the first postoperative night after undergoing urgent, uncomplicated surgery on a left lower leg fracture under general anesthesia. The night nurse reports that the patient was very anxious and had already received Tylenol i.v. for pain. However, she/he still reported diffuse pain in her/his upper body and nausea. The patient is a smoker and had asked when she/he was allowed to get up and smoke. Previous illnesses: Asthma (mild, only medication as needed), art. Hypertension, nicotine abuse, mild obesity, osteoarthritis of the right knee.

- What thought first crosses your mind? (open-ended)
- What is your medical evaluation? (open-ended)
- Which two differential diagnoses are you considering? (open-ended)
- What would you do next? Remember, it’s the middle of the night:
  ○ Reassure the patient and wait.
  ○ Reassure the patient and order stronger pain medication.
  ○ Initiate diagnostics.
- Which diagnostic studies do you initiate? (open-ended)
- You decide to examine the patient: He/she has cold sweats and has dyspnea. What would you do next? (open-ended)
- Cross your heart: the patient in the case study had a postoperative heart attack. Do you think that you underestimate cardiovascular events in female patients? (yes/no)
- Why do you think that cardiovascular events are generally underestimated by physicians? (open-ended)
- After menopause, the incidence of cardiovascular disease increases significantly in women because the protective effects of estrogen disappear. Do you have any idea how awareness for this phenomenon can be improved among physicians? (open-ended)

(The original survey can be requested by the corresponding authors).
